# Epidemiological profile of the polytraumatized patient: comparison between patients from an ICU in europe and an ICU in south america

**DOI:** 10.1186/2197-425X-3-S1-A365

**Published:** 2015-10-01

**Authors:** J Gonzalez Londoño, C Murcia Gubianas, C Vera Ching, C Cadavid, M Morales Pedrosa, JM Sirvent

**Affiliations:** Intensive Care Unit, University Hospital Dr Josep Trueta, Girona, Spain; Intensive Care Unit, Pablo Tobón Uribe Hospital, Medellin, Colombia

## Objectives

To compare the demographic characteristics, type and mechanism of trauma, need of interventions and survival of two cohorts of trauma patients in an Intensive Care Unit (ICU) of a tertiary hospital in Spain (HUJT) and another ICU of similar characteristics in Colombia (HPTU).

## Methods

Descriptive and comparative study of polytraumatized patients admitted to two different ICUs. The Spanish unit is an 18 bed general ICU, and trauma referral centre for the region of Gerona. The Colombian ICU has 19 beds dedicated mainly to trauma patients. Both hospitals have the same level of complexity, and resources are similar. The records of 151 polytraumatized patients between January 1 and June 30 of 2014 were reviewed, of which 32 came from the Spanish ICU and 119 from the Colombian one. A database where demographic characteristics, severity scores (ISS, APACHE II), location and etiology of trauma, surgery requirement and survival were recorded. Statistical analysis was performed with the *t* Student test and Pearson Chi -square accordingly.

## Results

From the demographic characteristics, Colombian patients were younger compared to Spain (37.1 ± 16.7 vs. 52.2 ± 20.7 years; p = 0.001). In both hospitals, the proportion of male patients was higher, but this difference was higher in Colombia with 82.4% of male patients; p = 0.02. The main mechanism of injury in both Colombia and Spain were traffic accidents, however while in Colombia violent injuries occupy the 2nd place (26.1%) in Spain they are the least common mechanism (3.1%); p = 0.005. Another difference between the two populations is the need for surgery, which is performed in Colombia in a greater percentage (69.7% vs. 34.4%); p = 0.0001. We found no significant differences between days of ICU admission, the ISS and APACHE II; we also found no differences in mortality.

## Conclusions

In comparison, patients admitted to the Colombian ICU are younger with a higher predominance of males and require surgery more often. On the other hand, and surprisingly, we found no statistically significant differences in the days of ICU admission, the ISS or APACHE II or mortality. It is likely that the differences are related to the mechanism of trauma, due to the higher prevalence of injuries due to violence in Colombia.

